# Statistical Dynamics of Flowing Red Blood Cells by Morphological Image Processing

**DOI:** 10.1371/journal.pcbi.1000288

**Published:** 2009-02-13

**Authors:** John M. Higgins, David T. Eddington, Sangeeta N. Bhatia, L. Mahadevan

**Affiliations:** 1 School of Engineering and Applied Sciences, Harvard University, Cambridge, Massachusetts, United States of America; 2 Department of Pathology, Brigham and Women's Hospital, Harvard Medical School, Boston, Massachusetts, United States of America; 3 Division of Health Sciences and Technology, Massachusetts Institute of Technology, Cambridge, Massachusetts, United States of America; 4 Department of Medicine, Brigham and Women's Hospital, Harvard Medical School, Boston, Massachusetts, United States of America; 5 Department of Electrical Engineering and Computer Science, Massachusetts Institute of Technology, Cambridge, Massachusetts, United States of America; 6 Department of Systems Biology, Harvard Medical School, Boston, Massachusetts, United States of America; City College of New York, United States of America

## Abstract

Blood is a dense suspension of soft non-Brownian cells of unique importance. Physiological blood flow involves complex interactions of blood cells with each other and with the environment due to the combined effects of varying cell concentration, cell morphology, cell rheology, and confinement. We analyze these interactions using computational morphological image analysis and machine learning algorithms to quantify the non-equilibrium fluctuations of cellular velocities in a minimal, quasi-two-dimensional microfluidic setting that enables high-resolution spatio-temporal measurements of blood cell flow. In particular, we measure the effective hydrodynamic diffusivity of blood cells and analyze its relationship to macroscopic properties such as bulk flow velocity and density. We also use the effective suspension temperature to distinguish the flow of normal red blood cells and pathological sickled red blood cells and suggest that this temperature may help to characterize the propensity for stasis in Virchow's Triad of blood clotting and thrombosis.

## Author Summary

Viewed from a distance, flowing blood looks like a uniform fluid, but up close the cells in the blood change their position and speed somewhat heterogeneously. These individual cell movements may play a role in the physiology and pathophysiology of nutrient and gas transport, clotting, and diseases where normal processes go wrong. To characterize these random motions, we need to follow individual cells in a very crowded suspension—cells usually occupy more than one-third of the volume in blood. We have developed computer software that can separate individual cells in a crowd and track them as they flow. We use this software to analyze blood flow at the level of the cell and find new and possibly important differences between the blood from healthy patients and the blood from patients with sickle cell disease, a disorder in which blood cells become stiff and often stop flowing. We provide evidence that blood from patients with sickle cell disease shows decreased random cellular motions and suggest that this difference may provide a physical basis for the increased risk of occlusion in sickle cell disease.

## Introduction

Red blood cells are the major component of blood and with a radius of ∼4 µm and a thickness of ∼1–2 µm are sufficiently large that the effects of thermal fluctuations are typically negligible, i.e. their equilibrium diffusivity is very small (
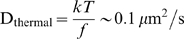
 where *f* is the viscous drag coefficient for a flat disk with radius 4 µm in water at room temperature [1]). However, when suspensions of these soft cells are driven by pressure gradients and/or subject to shear, complex multi-particle interactions give rise to local concentration and velocity gradients which then drive fluctuating particle movements [2]–[4]. Nearly all studies of whole blood to date focus on only the mean flow properties, with few notable exceptions [5]. Since the rheology of suspensions in general is largely determined by the dynamically evolving microstructure of the suspended particles [6], it is essential to measure both the dynamics of individual cells and the collective dynamics of cells in order to understand how the microscopic parameters and processes are related to larger scale phenomena such as jamming and clotting. We complement the large body of work characterizing the flow of sheared and sedimenting rigid particulate suspensions [7]–[11] and here study the statistical dynamics of pressure-driven soft concentrated suspensions while making connections to human physiology and disease. In particular, we provide quantitative evidence that there is heterogeneity in cellular velocity and density. This heterogeneity may play a role in the slow flow or stasis that can lead to the collective physiological and pathological processes of coagulation or thrombosis, as Virchow noted more than 100 years ago [12].

**Figure 1:**
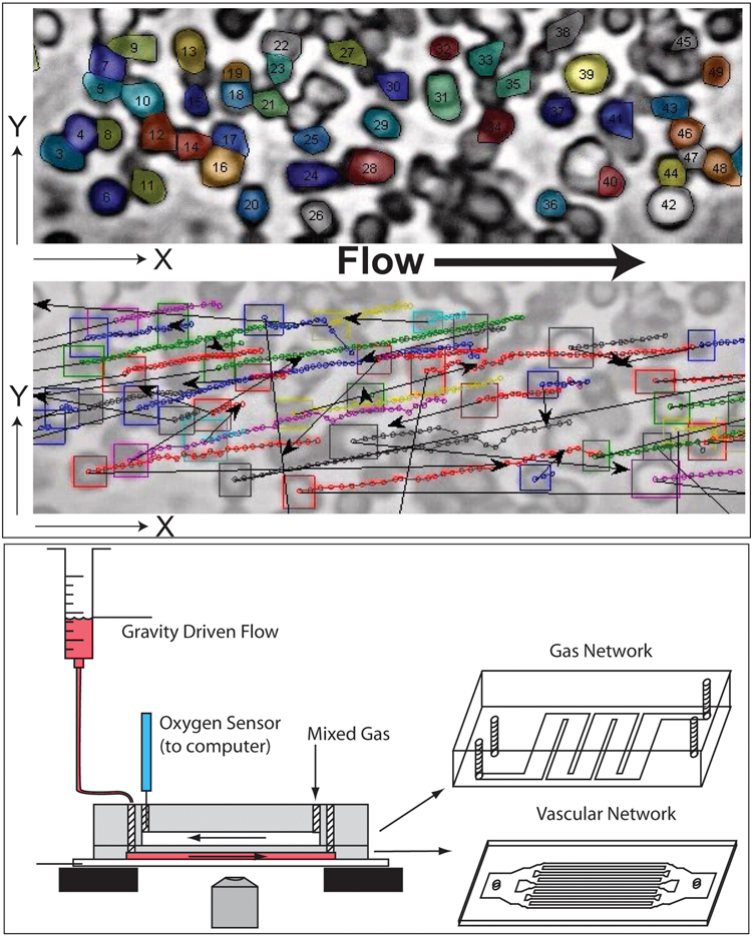
Cell tracking and experimental setup. The top panel shows a sample tracking image. Cells are segmented using morphological criteria and are tracked from frame to frame. The middle panel shows a subset of tracked cells, each with a bounding box. Each cell has a series of small color circles projecting from its centroid showing the subsequent trajectory. The black arrows represent that particular cell's velocity fluctuation relative to the median, with magnitude amplified by 4 for visualization. The bottom panel shows the experimental setup which is described in detail in [14] (see Videos S1, S2, S3, S4, S5, S6, S7, S8 for more detail).

**Figure 2:**
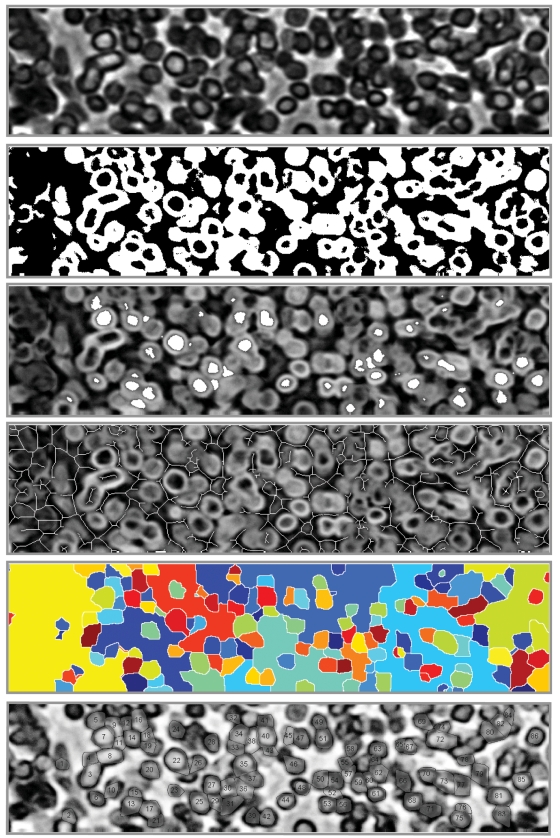
Snapshots of the segmentation process for a single video frame. See Methods for more detail. From top to bottom: 1. Raw video frame; 2. Thresholded binary version; 3. Foreground markers; 4. Background markers; 5. Marker-controlled watershed transformation; 6. Segmented objects filtered by size and shape. See Videos S1, S2, S3, S4, S5, S6, S7, S8 for additional detail.

To investigate the short-time dynamics of flowing red blood cells we develop and use computational image processing [13] and machine learning algorithms to segment and track individual blood cells in videos captured at high spatial and temporal resolution in a microfluidic device (Figures 1 and 2 and Videos S1, S2, S3, S4, S5, S6, S7, S8). We measure individual cell trajectories comprised of more than 25 million steps across more than 500,000 video frames. These measurements enable us to ask and answer questions about the variability of velocity fluctuations at the scale of individual normal and sickled red blood cells with variable shape and rigidity. We quantify the effect of bulk flow velocity and density on the microscopic velocity fluctuations, and the role of collective behavior under pathological conditions which alter these properties.

We utilized microfluidic devices with cross-sectional area of 250 µm×12 µm, similar to the devices used to characterize the phase diagram for vaso-occlusion in an *in vitro* model of sickle cell disease [14]. The 12 µm dimension of the microfluidic channels along one axis confines the cell movements in this direction; indeed the range of motion is already hydrodynamically limited by the Fahraeus effect [15]. The primary advantage of this quasi-two-dimensional experimental geometry is the ability to visualize the cells easily, because any significant increase in the size of the channel in this direction would make the cell tracking impossible. This small dimension changes the dynamics as compared to those of cells moving through large circular channels, owing to the effects of the relatively large shear rates in the narrow dimension and our inability to measure fluctuations along this axis, but our system nevertheless enables the characterization and measurement of the quasi-two-dimensional statistical dynamics of both normal and pathological blood flow with very high time and spatial resolution. We chose a set of device and blood parameters relevant to human physiology and pathology in the microcirculation associated with capillaries and post-capillary venules. We derived our quasi-two-dimensional data from the middle fifth of the 250 µm-high channel, where the narrow 12 µm thickness provides the only significant shearing direction, and this shear rate (∼10/sec) is in the physiological range for the microcirculation [15].

## Results

**Figure 3:**
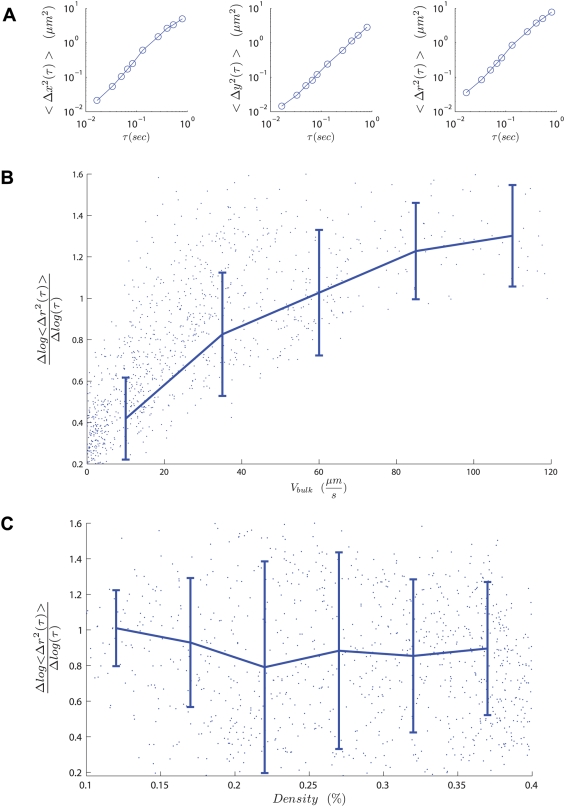
Cellular-scale dynamics. The top panel (a) shows average fluctuations in squared cellular displacement as a function of time (e.g., 〈Δr^2^(τ)〉) with x- and y-axes defined in the top panel of Figure 1. The middle panel (b) shows the nature of the collective microscopic dynamics characterized by 
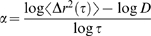
 (see text). The dynamics are diffusive for V_bulk_>50 µm/s. Error bars show medians and standard deviations for binned data. The bottom panel (c) compares cellular-scale dynamics to cellular volume fraction and shows that density variation in this range has no effect on the nature of cellular scale dynamics.

Figure 3a quantifies the planar fluctuations of individual blood cells in terms of the mean-squared displacement, 〈Δr^2^(τ)〉 = 〈(r_bulk_(τ)−r_cell_(τ))^2^〉 where 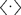 denotes a spatial average, and shows that 〈Δr^2^(τ)〉 = Dτ, with an effective diffusion constant D much larger than the equilibrium diffusivity (∼0.1 µm^2^/s). (See Videos S1, S2, S3, S4, S5, S6, S7, S8 for examples of this diffusive behavior.) Thus movement of a cell in relation to the bulk at one instant becomes rapidly decorrelated with its subsequent movement, except over very short times relative to the time of interaction between cells. 〈Δr^2^(τ)〉 is roughly isotropic at shorter times, and then anisotropic at longer times with fluctuations parallel to the direction of flow 50% larger than perpendicular to it, a finding which is qualitatively consistent with observations of sheared and sedimenting rigid particulate suspensions [3],[16]. This diffusive behaviour is itself dynamical in its origin, being driven by the relative flow of fluid and cells and the boundary. To understand this dependence, we also plotted in Figure 3b the evolution of the scaling exponent 
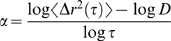
 as a function of the bulk flow velocity (V_bulk_) and red blood cell concentration for more than 700 different experiments with different blood samples. We find that an increase in V_bulk_ from rest to about 50 µm/s is associated with a change in dynamics from stationary through sub-diffusive to diffusive. However, over the pathophysiologically relevant range of densities studied (15%–45%) there is no consistent effect on the nature of the statistical cell dynamics. Figure 3b shows significant variation in this dynamical process, and only by combining measurements of a large number of cell trajectories are we able to see that the curve flattens with increasing V_bulk_ as α approaches 1.0. Further, in Figure 3c we show that 〈α〉∼1.0, providing additional support for the conclusion that the typical flow is diffusive.

**Figure 4:**
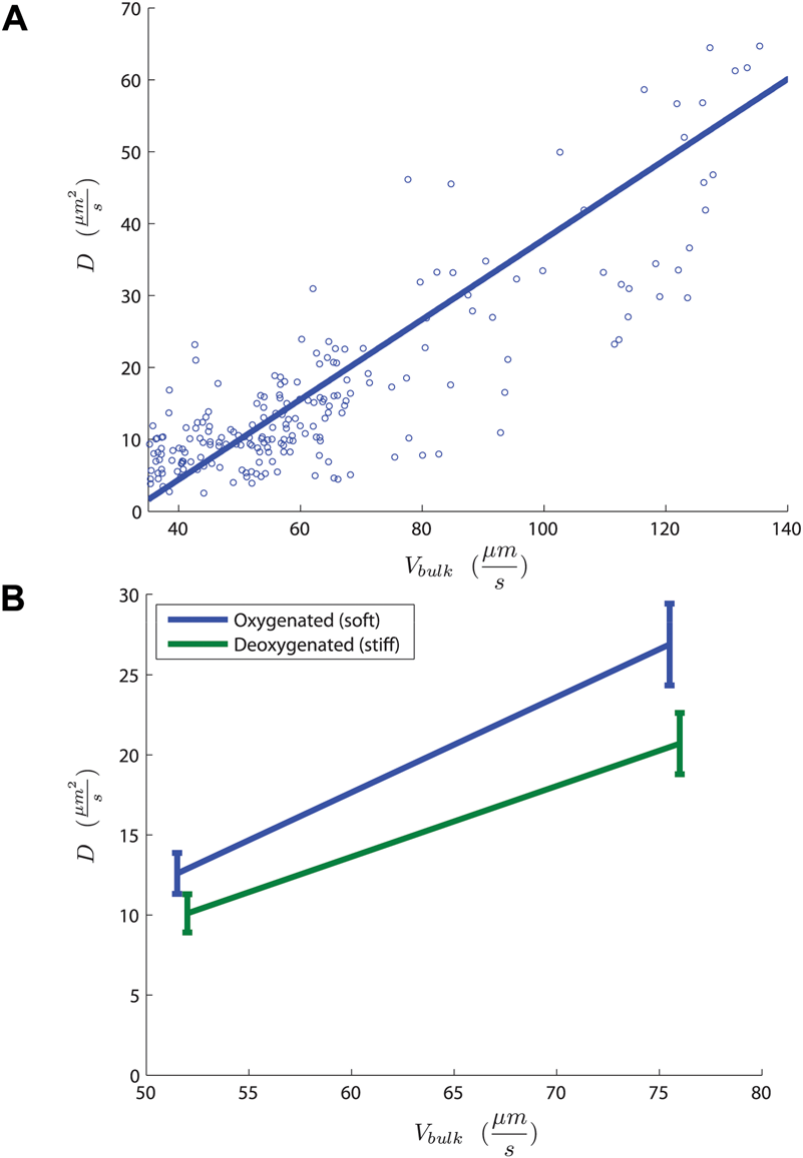
Shear-induced diffusion coefficients. The top panel (a) shows the hydrodynamic diffusion coefficient D as a function of the bulk flow velocity V_bulk_ for flows fast enough for the diffusive behavior to be recovered, i.e. V_bulk_>∼50 µm/s based on Figure 3. The bottom panel (b) compares this relationship for soft oxygenated sickle cells and stiff deoxygenated sickle cells where we see that D_deoxygenated_<D_oxygenated_. Error bars show medians and standard error for binned data.

A diffusive process has a characteristic length scale λ corresponding to the mean free path that a cell travels before an interaction, and a characteristic time scale corresponding to the time between these interactions, typically given by the inverse of local shear rate 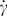, at the low Reynolds numbers typical of microvasculature flows *in vivo* as well as in our experiments (where *Re* = O(0.01)). Then the effective diffusivity scales as 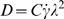, where *C* is a dimensionless constant which will depend on microscopic properties such as cell shape and rigidity. There are three length scales in the problem that can determine the effective diffusive length scale λ: cell size, cell separation, and cell distance from the boundary. Different length scales will dominate in different limits of density, geometry, and cell size, as a cell will travel only a fraction of the inter-cellular distance before it interacts with another cell or a boundary. In the unconfined limit where the boundary is infinitely far away, the only characteristic scale is the cell size so that 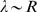, and 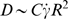. This dilute limit has received the most attention to date [2],[4], but is far from the soft, dense, and confined suspensions we study. The two remaining origins for this characteristic scale are: (i) the distance between cells (about 3 µm at a two-dimensional density of 33%) which is comparable to and even smaller than the cell size; (ii) the small height of our channel, 12 µm, which implies that the discoid red blood cells interact with the wall. The cells are typically oriented with their discoid faces perpendicular to the smallest dimension of the channel. The strong local shear (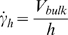, where *2h* is the channel height) relative to the wall leads to an effective diffusivity 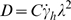, where 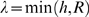. As has previously been shown [4],[6],[16],[17], a velocity gradient can lead to particle interactions and rearrangements in all three principal directions particularly when the shapes of the particles are non-spherical as here. This is particularly true in our study because the particles (cells) are disc-like and deformable, so that the combination of shape anisotropy and the generation of normal forces via tangential interaction in soft contact can lead to diffusive motions in the measurement plane [18]. In Figure 4a, we show this diffusive behaviour for V_bulk_ >∼50 µm/s. The measured D≈8 µm^2^/s, and 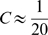 for λ ∼ 3 µm. By sampling over times longer than 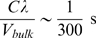, our measurements reach far enough into the asymptotic behavior of the dynamics to characterize this diffusive process. Over shorter times, we expect a mixture of diffusive and ballistic dynamics, though this effect in our results is dominated by the fact that extremely small displacements are below our analytic sensitivity and appear as stasis. In addition, cell velocities fluctuate because of the localized spatio-temporal fluctuations in shear rate, i.e., 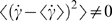. (See Videos S1, S2, S3, S4, S5, S6, S7, S8.) These shear rate fluctuations could potentially also contribute to the effective diffusivity of the cells, but here we limit ourselves to the simplest mean field picture that ignores the fluctuations in the shear rate itself.

To assess the relative role of microscopic determinants such as cell shape and stiffness on this diffusive process, we investigated the behavior of blood cells from patients with sickle cell disease. Red blood cells from these patients become stiff in deoxygenated environments as a result of the polymerization of a variant hemoglobin molecule [19], resulting in a dramatic increase in the risk of sudden vaso-occlusive events with a poorly understood mechanism [20]. In Figure 4b, we plot *D* versus *V_bulk_* for oxygenated and deoxygenated sickle cell blood and see that for a given bulk flow rate, the stiffer cells have a smaller diffusivity. Since 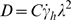, our results therefore imply that *C*_deoxygenated_<*C*_oxygenated_, i.e., the stiffness of the cells influences the dynamics of a pressure-driven suspension independent of *V_bulk_*, likely due to changes in the nature of the interactions of cells with each other, with the channel walls, or with the plasma velocity gradients. The tangential and normal forces between two fluid-lubricated soft moving objects is a complex function of shape, separation, stiffness, relative velocity, and fluid viscosity. Tangential interactions between soft cells lead to normal forces that push the cells away from each other, thus reducing the friction between them [18]. Since the effective diffusion coefficient of this driven system is inversely proportional to the frictional drag, we expect the diffusion coefficient for the stiffer cells to be smaller than that for soft oxygenated cells when the flow velocity is held constant, as is observed.

## Discussion

**Figure 5:**
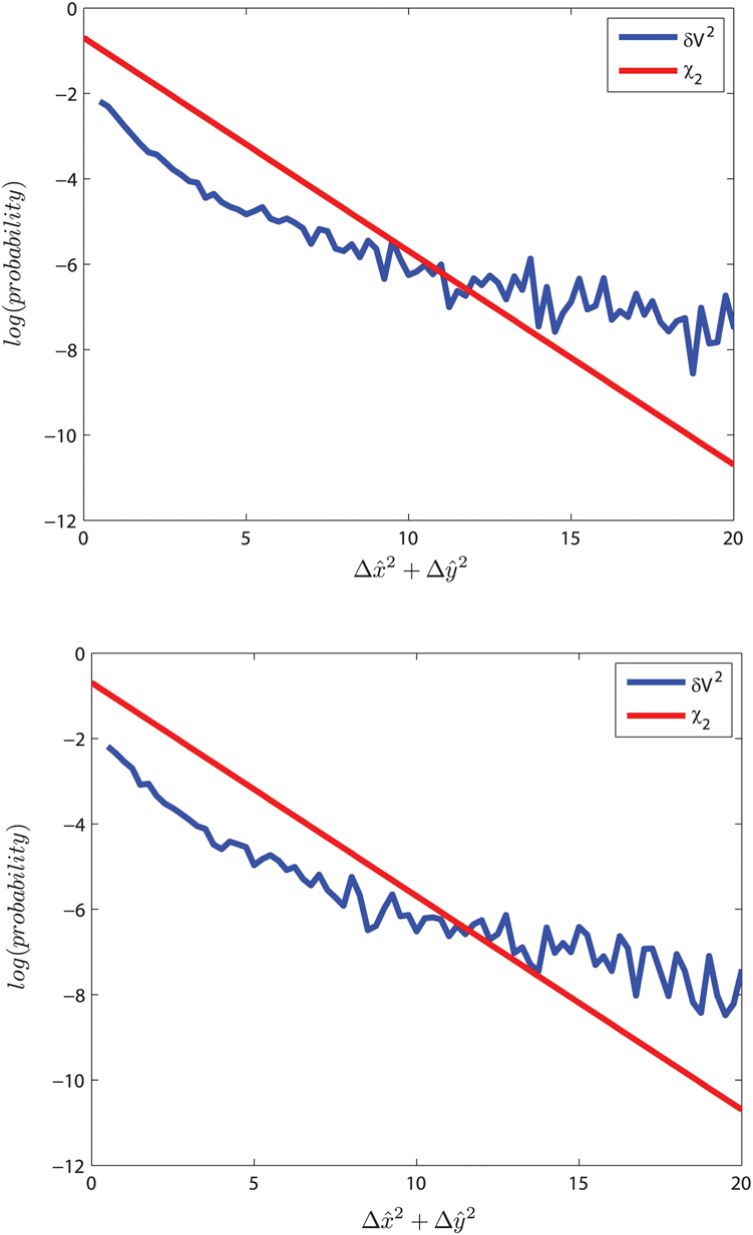
Cellular velocity fluctuations as an effective temperature. These two panels compare probability distribution functions for normalized squared velocity fluctuations from two different experiments with chi-squared distributions with 2 degrees of freedom. 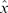 is normalized with mean 0 and standard deviation 1, and x- and y-axes are defined in the top panel of Figure 1. This comparison shows that blood flow has an effective suspension temperature with longer tails as a result of the non-equilibrium nature of the pressure-driven system.

Hydrodynamic interactions between red blood cells lead to velocity fluctuations and diffusive dynamics of the individual cells. Changes in *V_bulk_* or cellular stiffness alter *D* and therefore control the magnitude of velocity fluctuations. Cellular velocity fluctuations are quantified by their mean square, 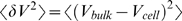, which may be interpreted in the language of the statistical physics of driven suspensions [16],[21] as an effective suspension temperature. Just as thermal temperature reflects the mean squared molecular velocity fluctuation, the suspension temperature reflects the mean squared cellular velocity fluctuation. This temperature will then change with *V_bulk_* as well as with particle stiffness. Slower flows will have lower effective suspension temperature, as will flows of stiffer particles. In Figure 5, we show the measured probability distribution of δV^2^ for two different flow experiments and see that it has longer tails than an equilibrium Maxwell-Boltzmann distribution owing to the non-equilibrium nature of the system, consistent with observations in physical suspensions [3],[10]. We may nevertheless use the crude analogy of an effective temperature to characterize “hot” blood flow which has increased 〈δV^2^〉 and is also less likely to coagulate or “freeze” than is a “cold” blood flow where cells are not fluctuating and local stasis is more likely to arise and to persist. Virchow's Triad characterizes the conditions leading to thrombosis as stasis, endothelial dysfunction, and hypercoagulability [12] and our results offer one possible explanation for why pathological blood with stiffer cells and smaller cellular velocity fluctuations will occlude at flow rates where normal blood will not.

In conclusion, we have identified random walk-like behavior for pressure-driven dense suspensions of soft particles in quasi-two-dimensional confinement which we quantify in terms of cellular velocity fluctuations as a function of blood flow rate, shape, and stiffness. Our results suggest that these fluctuations may be involved in the collective pathophysiological processes of occlusion and thrombosis, both of which are strongly heterogeneous in space and time. While simple scaling ideas are suggestive, a well-defined microscopic mechanism for this process remains to be established.

## Methods

### Ethics Statement

This study was conducted according to the principles expressed in the Declaration of Helsinki. The study was approved by the Institutional Review Board of Partners Healthcare Systems (2006-P-000066). All patients provided written informed consent for the collection of samples and subsequent analysis.

### Blood Flow Video Acquisition

Videos were captured of blood flowing in microfluidic devices under controlled oxygen concentration. Microfluidic fabrication and blood sample collection and handling are described in detail elsewhere [14]. Blood flowed through channels with cross-sectional dimension of 250×12 µm and was driven by a constant pressure head. A juxtaposted network of gas channels allowed control over the oxygen concentration within the blood channel network. Blood samples were collected in EDTA vacutainers and had hematocrit ranging from 18% to 38%. By changing oxygen concentration *in situ*, we were able to compare the oxygenated and deoxygenated behavior of the same sample and largely control for any differential contributions of the plasma. Videos were captured at a rate of 60 frames per second, with a resolution of about 6 pixels per micron. (See Videos S1, S2, S3, S4, S5, S6, S7, S8 for examples.) We note that the rapid rate of deoxygenation in our studies results in little change in shape for most cells, consistent with existing understanding of heterogeneous hemoglobin polymerization, while the magnitude of the change in stiffness is expected to be more independent of deoxygenation rate [19],[22].

### Blood Cell Image Segmentation

We developed morphological image processing algorithms to identify a significant fraction of the cells in captured frames of video. See Figure 2 for examples of the segmentation approach. All software was written in MATLAB (The MathWorks, Natick, Mass.). These algorithms implement marker-controlled watershed segmentation, described in detail in reference 13. Marker images were computed by identifying annular and filled cells of heuristically-determined sizes and shapes.

Annular cells were defined as fillable holes not touching the border. Markers for these annuli were created by subtracting border-contacting high-intensity regions and performing morphologic reconstruction on the result. This reconstruction operation used a marker image that was morphologically opened with a 5 µm line segment oriented in increments of 45 degrees. The reconstruction was then subtracted from the border-cleared image. The final result was dilated using a disk with radius 0.2 µm. Filled cells were defined using granulometry with a circular structuring element of radius 2 µm. Markers for these cells were selected using two transformations of this opened image: the distance transformation of the thresholded binary image followed by the h-maxima transformation with a height of 3.

Background pixels were identified by the skeletonization of a thresholded binary image. Previously determined cell markers were added to the binary image. The result was eroded using a disk with radius 0.5 µm. The skeletonization of this erosion was the background marker image. Foreground and background markers were used to impose minima on the intensity gradient of the original image after background subtraction and histogram equalization. The watershed transformation was then applied to the gradient of the intensity image.

The watershed catchment basins, or blobs, were then filtered heuristically by size, shape, and orientation of the objects' convex hulls. First-pass thresholds were determined empirically by manually segmenting several video frames in Adobe Photoshop. Initial size limits were total convex hull area between 5 and 50 µm^2^. A measure of convex hull circularity was calculated by comparing the effective radius based on the object area to the effective radius based on the object's perimeter. A circle has a ratio of 1. All other objects have ratios less than 1. The initial circularity threshold was set at 0.6. After an initial filtering process, video frames were re-filtered using thresholds for all morphologic characteristics based on the mean convex hull metrics with allowed variation of twice the standard deviation.

### Blood Cell Tracking Between Frames

We then developed machine learning algorithms to track these segmented cells from frame to frame and to compute velocities for individual cells. For each object segmented in each video frame, potential “child” cells were iteratively identified in the subsequent frame and ranked by changes in size, shape, and displacement. Child cells were reassigned if a better “parent” cell was identified. Maximum changes in x- and y-displacement were calculated based on apparent flow rates. Y displacement was limited to 600 µm/s in either direction, and x displacement was limited to 1200 µm/s. Maximum changes in area, perimeter length, and eccentricity were determined by manual tracking of several video frames in Adobe Photoshop as part of a validation check on the tracking algorithm. Area was initially allowed to vary by 50%, perimeter by 50%, and eccentricity by 60%.

After all cells in a frame were tracked or determined to be un-trackable, the median inter-frame displacement was computed for all tracked objects. Any tracking events representing displacements that were five times greater than the maximum of the median or the analytic sensitivity threshold (1 µm) were excluded, and the whole frame was retracked with this tighter displacement threshold.

Tracking events which represented the extension of existing trajectories were rejected if they represented a change in cell velocity greater than twice the maximum of the median frame displacement or an analytic sensitivity threshold. After excluding these inconsistent tracking events, the whole video frame was retracked iteratively until no trajectory extensions exceeded this threshold.

### Assessment of Calculated Cell Velocity

Our measured cell velocities were based on more than 25 million displacements calculated across more than 500,000 video frames. We improved and measured the accuracy of our cell velocity measurements a number of different ways, including manual segmentation by an observer of selected video frames and manual tracking by an observer of selected of cells from frame to frame. Inaccuracies in cell velocity measurements can be separated into two categories: errors in the location of a cell, and errors in the assignment of a tracking event for two identified cells. We took a series of steps to reduce the magnitude and bias of this noise and to ensure that it does not influence our results.

#### Reducing noise in cell location

The first type of inaccuracy comes from the need to assign a single pixel location to each cell. We chose the centroid of each segmented pixel blob. Because of random variation in image intensity due to lighting and movement of cells out of the focal plane, the calculated centroid for a given cell cannot be established with absolute precision. We estimated this uncertainty first by optimizing our segmentation algorithm based on several video frames segmented manually in Adobe Photoshop, and second by manually tracking several dozen trajectories and comparing our manual cell positions with those of the segmentation and tracking algorithms. We determined that the analytic resolution of the combined segmentation and tracking algorithms was at least 5 pixels (∼1 µm), meaning that the true location of a cell identified in a video frame could vary at least 5 pixels in any direction from the calculated position. This analytic sensitivity was then used to form a tolerance in heuristic cell tracking.

#### Reducing noise from false positive tracking events

The second type of inaccuracy comes from the false positive linking of segmented blobs in successive video frames. These false positive tracking events could involve actual but distinct cells as well as spurious cells. In any video frame, there is a chance that non-cellular regions (e.g., circular regions of plasma bordered by cells) will have an intensity pattern similar enough to that of a cell to be identified as a cell. If these regions persist from one frame to the next, they may be falsely identified as a tracked cell, and their “velocities” will degrade the accuracy of our results. We took steps to prevent the introduction of such false positives in our data, and we took further steps to reduce the impact these false positives.

To prevent the introduction of false positives in our data, we first optimized our segmentation algorithm to minimize the introduction of false positives. We manually segmented several video frames using Adobe Photoshop and optimized our cellular segmentation algorithms by comparing calculated segmentation functions to manual segmentation functions. We then filtered image blobs by morphologic characteristics including area, perimeter, orientation, eccentricity, and shape. Thresholds for this heuristic filtering process were developed from the analysis of several manually-segmented video frames.

To reduce the impact of false positives and to improve the accuracy of the tracking algorithm, we used several heuristics. Tracking events were identified by evaluating cells in two passes. In the first pass, wide tolerances were used to identify likely tracking events without bias. The median of these displacements was then used to form tighter tolerances for a second pass. This second pass removed any tracking events which required displacements greater than five times the median displacement and five times the analytic sensitivity in the x- or y-direction. If a tracking event was added to an existing trajectory, the integrity or consistency of that trajectory was assessed. We excluded any change in displacement relative to the median that was greater than twice the median in either direction and twice the analytic sensitivity.

Velocities calculated for all processed videos were then assessed by comparing with tracking results for random composite videos. We assembled videos with successive frames randomly stitched together from different videos or from the same video but sampled from time points such that no cell would appear on two consecutive frames. Any tracking events identified in these videos were false positives. We created dozens of these videos and used them to estimate the false positive rate of our tracking logic. These random videos rarely yielded more than 10 tracking events between successive frames. We doubled this number and used a conservative threshold of 20 tracking events. We excluded any video from our analysis if a single pair of successive frames yielded fewer than 20 tracking events.

False positive tracking events were less likely to persist in multiple-step trajectories. For well-tracked videos, most of the shorter trajectories would persist as longer trajectories. We established minimum quality thresholds for the number of longer trajectories as a proportion of shorter trajectories. If too few of the shorter trajectories were successfully tracked for more frames, the videos were excluded from the analysis.

The image processing errors for each frame are likely to be independent from one frame to the next. The true velocity fluctuations, however, are likely to be correlated from frame to frame over very short times. We can therefore look at these measured fluctuations in velocity between different cells over increasing time intervals and confirm that they decrease as they are averaged over more and more frames. We know that over long times, there is a well-defined bulk flow velocity. Individual cells do not zoom ahead of the bulk over long times, nor do they stop in the middle of the stream for significant periods of time. Over long times, the fluctuations of individual cell velocities must therefore regress to zero, and the coefficient of variation measured over these long times will tend to zero, as is the case for these instances of normal blood in steady flow. The decreasing coefficient of variation therefore supports the validity of these velocity measurements.

The segmentation and tracking algorithms work best for cells that are isolated, appear in the focal plane, and generate a sharp phase contrast in the microscope. Cells in this subset which retain these characteristics across several frames will contribute very accurate velocity measurements. One can therefore be very confident that the median cell velocities calculated for cells with long trajectories will be valid. We can then compare cumulative displacements of cells with long tracking trajectories to overall cumulative displacements to assess the validity of tracking information derived from a given video.

#### Assessing noise in final data

Finally, in our data analysis, we compared our overall results to those for subsets of our data consisting of velocities calculated only from longer trajectories as compared to velocities calculated for shorter trajectories. We reasoned that the noise in our data set remaining after data processing is more prevalent in the shorter trajectories. The effects of limited analytic sensitivity will average out over long trajectories, and false positive segmentation and tracking events are very unlikely to persist across several frames. We re-ran our analysis using these reduced data sets and confirmed our reported findings.

### Measurement of Two-Dimensional Cell Density

We measured projected cell density first by thresholding grayscale intensity images using the MATLAB graythresh function. We then combined this thresholded image with the foreground cell markers calculated by our segmentation algorithm. Under steady state conditions, we would expect this density calculation to be relatively stable.

Previous studies have reported a coefficient of variation for hematocrit of 3% due to biological variation, and another 3% due to analytic variation achieved with commonly used automated hematologic analyzers [23]. These automated analyzers work with typical volumes of (20,000 cells*1/0.4 total volume/cell volume*80 µm^3^ cell volume/cell = 4×10^6^ µm^3^), which is about 100 times larger than the volume projected in a typical video frame.

The relationship between an actual three-dimensional volumetric density and a projected two-dimensional density depends on the orientation of the red blood cells and the depth of the flow chamber in the direction of the projection. Under steady state conditions, our density measure is stable over time with a coefficient of variation typically between 10% and 25%.

## Supporting Information

Video S1 A 3-second video of sickle cell blood captured at 60 frames per second flowing in 10% oxygen at about 53 µm/s.
(8.16 MB CDR)

Video S2 Video S1 with segmented cells highlighted in color. Color will stay constant if the cell is tracked from one frame to the next.
(8.14 MB CDR)

Video S3 Video S1 showing all tracked cell trajectories greater than 4 frames long. Each tracked cell also has a black line showing 4 times the velocity deviation vector with respect to the bulk.
(8.17 MB CDR)

Video S4 Video S1 showing a translating rectangular frame of reference. The rectangle moves with the bulk in the bottom panel, and this translating frame is the frame of reference in the top panel.
(2.90 MB CDR)

Video S5 A 3-second video of sickle cell blood captured at 60 frames per second flowing in 0% oxygen at about 59 µm/s.
(8.15 MB CDR)

Video S6 Video S5 with segmented cells highlighted in color. Color will stay constant if the cell is tracked from one frame to the next.
(8.15 MB CDR)

Video S7 Video S5 showing all tracked cell trajectories greater than 4 frames long. Each tracked cell also has a black line showing 4 times the velocity deviation vector with respect to the bulk.
(8.16 MB CDR)

Video S8 Video S5 showing a translating rectangular frame of reference. The rectangle moves with the bulk in the bottom panel, and this translating frame is the frame of reference in the top panel.
(2.68 MB CDR)
